# An early sex difference in the relation between mental rotation and object preference

**DOI:** 10.3389/fpsyg.2015.00558

**Published:** 2015-05-08

**Authors:** Jillian E. Lauer, Hallie B. Udelson, Sung O. Jeon, Stella F. Lourenco

**Affiliations:** ^1^Department of Psychology, Emory University, Atlanta, GA, USA; ^2^School of Medicine & Dentistry, University of Rochester, Rochester, NY, USA

**Keywords:** mental rotation, object preference, visual attention, infancy, sex differences

## Abstract

Accumulating evidence suggests that males outperform females on mental rotation tasks as early as infancy. Sex differences in object preference have also been shown to emerge early in development and precede sex-typed play in childhood. Although research with adults and older children is suggestive of a relationship between play preferences and visuospatial abilities, including mental rotation, little is known about the developmental origins of this relationship. The present study compared mental rotation ability and object preference in 6- to 13-month-old infants. We used a novel paradigm to examine individual differences in infants’ mental rotation abilities as well as their differential preference for one of two sex-typed objects. A sex difference was found on both tasks, with boys showing an advantage in performance on the mental rotation task and exhibiting greater visual attention to the male-typed object (i.e., a toy truck) than to the female-typed object (i.e., a doll) in comparison to girls. Moreover, we found a relation between mental rotation and object preference that varied by sex. Greater visual interest in the male-typed object was related to greater mental rotation performance in boys, but not in girls. Possible explanations related to perceptual biases, prenatal androgen exposure, and experiential influences for this sex difference are discussed.

## Introduction

Humans rely on a variety of visuospatial processes to perform many of the activities essential to everyday life. A key component of visuospatial processing is mental rotation, which refers to the mental transformation of object representations. Shepard and Metzler (1971) first documented mental rotation in a seminal study in which adult participants were asked to judge whether pairs of figures depicted the same object in two different orientations or an object paired with its mirror image (also in different orientations). Objects were different when one figure was the mirror image of the other. In making this judgment, participants’ response times varied as a function of the angular disparity between the two figures, suggesting an analog between rotations in mental and physical space. Despite the importance of this early study, its small sample size prevented analyses of individual or group differences in performance. Later research, however, has pointed to substantial intra- and inter-group variability, including sex differences. On typical mental rotation tasks, men outperform women in adulthood (*d* = 0.66; for a meta-analysis, see Voyer et al., 1995). This male advantage is detectable, albeit smaller, in preschool-aged children (*d* = 0.25; Levine et al., 1999) and increases throughout childhood (Voyer et al., 1995). Accumulating evidence suggests that the ability to engage in mental rotation on tasks similar to that developed by Shepard and Metzler is related to achievement in science, technology, engineering, and mathematics (STEM) and may contribute to gender disparities in these fields (Casey et al., 1997; Ganley et al., 2014), underscoring the importance of understanding the developmental origins of both individual and group differences in this spatial ability.

Only a handful of studies have investigated mental rotation in very young children. In the first empirical investigation of this ability in infancy, Rochat and Hespos (1996; see also, Hespos and Rochat, 1997) examined 4- to 8-month-olds’ sensitivity to occluded rotational movement. Specifically, the authors examined whether infants’ looking times were consistent with the predicted trajectory of a T-shaped object, which rotated 180° from the top to the bottom of a screen. Critically, part of this rotation occurred behind an occluder such that the object’s movement could not be tracked perceptually. When the occluder was removed, the object appeared in either a probable or improbable orientation given its earlier trajectory. Across the age range tested, infants looked longer to the improbable orientation, suggesting they were able to predict the object’s trajectory by mentally rotating the object. Using a similar procedure, Frick and Möhring (2013) showed that 10-month-olds discriminate between rotating figures and their mirror images, indicating that infants possess mental rotation processes similar to those reported in older children and adults.

Also paralleling work in older samples, recent studies have reported a male advantage in performance on mental rotation tasks as early as 3 to 5 months of age. In one study, 5-month-old infants were habituated to a three-dimensional object rotating 240° in depth (Moore and Johnson, 2008). When infants were later presented with the same object or its mirror image rotating through a novel 120° arc (the remaining path of a 360° rotation), only boys showed greater looking times toward the mirror object. Girls did not discriminate between the two objects. In another study, 3- to 4-month-olds were familiarized with the numeral 1 in varying orientations along the picture plane and then presented with the familiarized stimulus and its mirror image in a previously unseen orientation (Quinn and Liben, 2008). Again, only boys preferred the mirrored stimulus; girls showed no preference for either object. In a later study, Quinn and Liben (2014) reported that this male advantage held across 3- to 10-month-olds. However, not all studies have found sex differences in this age range (Möhring and Frick, 2013; Schwarzer et al., 2013), emphasizing the need for additional research.

What might account for an early sex difference on mental rotation tasks? One theory proposes that variability in mental rotation ability relates to differential androgen exposure *in utero*. This theory is supported by findings showing that greater prenatal androgen levels predict greater mental rotation ability in healthy young girls (Grimshaw et al., 1995). Similarly, research on females with congenital adrenal hyperplasia (CAH), a genetic disorder associated with increased prenatal androgen exposure, has shown that these individuals have greater mental rotation abilities compared to typically developing girls (Resnick et al., 1986). Another theory posits that sex differences in mental rotation (and spatial abilities more generally) are the result of differential interest in and experience with spatially relevant toys. In support of this theory, Terlecki et al. (2008) showed that exposure to a spatially relevant computer game in adulthood increased performance on a standard mental rotation task (Vandenberg and Kuse, 1978), assessed using pre- and post-tests. Alexander (2006) found that adults’ visual interest in male- over female-typed toys was related to better performance on a visuospatial task, though no such relation existed between reported childhood experience with such toys and spatial ability in adulthood. Although these findings suggest a relationship between spatial activities and mental rotation, they do not account for sex differences in mental rotation in infancy, when spatial activities are likely minimal.

The findings discussed above demonstrate some efforts to understand the relationship between sex differences in mental rotation and spatial activities in adulthood, but there has been far less research on whether similar links exist earlier in development and no such studies, to our knowledge, have been conducted in infancy. It has been suggested that childhood participation in spatial activities (e.g., play with puzzles and blocks) is related to spatial abilities later in life (for meta-analysis, see Baenninger and Newcombe, 1989; Voyer et al., 2000), but studies in this area often rely on retrospective reports of childhood experience rather than concurrent measures, and do not generally distinguish between different spatial abilities such as mental rotation and navigation. Although infants are less exposed to spatial activities than older children and adults, sex differences in object preferences may be present from birth, as male, but not female, neonates exhibit a greater preference for mechanical objects over socially relevant stimuli (e.g., human faces; Connellan et al., 2000). The extent to which object preferences relate to mental rotation is unknown, however. Given that infants show sex differences in both mental rotation and object preference, the current study allowed us not only to extend previous work on the generalizability of early sex differences related to each, but also to examine the relationship between mental rotation and object preference. We gave 6- to 13-month-old infants a visuospatial task designed to tap mental rotation as well as a standard object preference task. Both tasks involved paired presentation of stimuli (two-alternative forced-choice) and measures of visual preference. The similarity in procedures and dependent variable, described in greater detail below, allowed for a direct comparison of individual differences across the two tasks.

**FIGURE 1:**
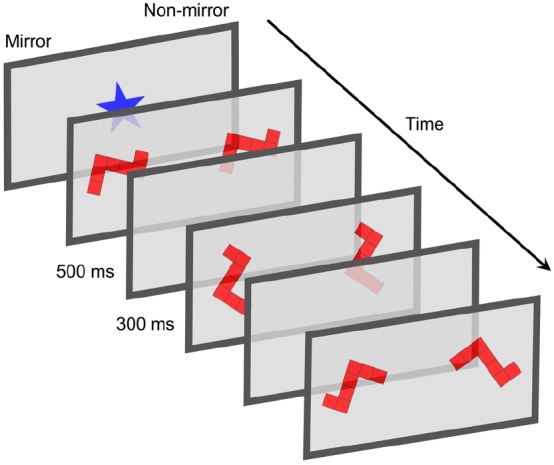
**Change detection paradigm used to assess mental rotation.** Two image streams were presented simultaneously on opposing sides of a frontal screen. Stimuli were presented for 500 ms and followed by an interstimulus interval (ISI) of 300 ms. The two streams were identical except that on every third presentation, the stimulus in the mirror stream consisted of the mirror image of the stimulus presented in the non-mirror stream. Figure not to scale.

Our visuospatial task was based on a “change detection” paradigm used originally by Ross-Sheehy et al. (2003) to assess infants’ visual short-term memory and adapted by Libertus and Brannon (2010) to measure individual differences in preverbal number representations. For our purposes, it was modified to capture infants’ sensitivity to visuospatial information, particularly the extent to which they are capable of mental rotation. Infants were presented with two image streams containing a figure that appeared in different orientations. The streams were identical except that in one, which we refer to as the “mirror stream,” the figure was mirrored across each trial (see Figure 1). The other stream contained no mirrored figure. During change detection tasks, infants are expected to exhibit greater attention to (i.e., longer looking times toward) the stream with greater change if they are able to detect the change, as infants prefer greater variability, or novelty, on this task (Ross-Sheehy et al., 2003; Libertus and Brannon, 2010). Here, preferential looking toward the mirror stream would suggest that infants recognized the novelty of the mirror image, which should be considered different than the stimuli in the non-mirror stream because the mirrored figure cannot be rotated into alignment with the others. As in standard mental rotation tasks, confining orientations to the picture plane differentiates the figure from its mirror image, a difference that one can confirm via mental rotation (see also Quinn and Liben, 2014).

Although the change detection paradigm is less common than other visual attention procedures such as habituation/dishabituation, we adopted it to assess mental rotation for two reasons. First, it does not include a familiarization phase designed to decrease attention as measured by a formal criterion (e.g., 50% decrease in looking times common in habituation/dishabituation paradigms). The lack of such a phase in this study was critical in order to minimize attrition across the administration of two tasks. We thus opted for a change detection paradigm that employs continuous presentation of stimuli without a separate habituation phase. Second, infant research is often constrained by paradigms that are compatible with the dependent variable of looking time. Use of different procedures (e.g., habituation/dishabituation vs. change detection) is thus important for testing the generalizability of findings, especially in light of inconsistent findings on sex differences in mental rotation (e.g., Moore and Johnson, 2011; Schwarzer et al., 2013).

## Materials and Methods

### Participants

Fifty-six healthy infants between the ages of 6 and 13 months participated in this study. There were an equal number of girls and boys, with no significant difference in age (girls: *M* = 9.71 months, SD = 1.70; boys: *M* = 10.54 months, SD = 1.88; *t*(54) = 1.74, *p* > 0.09). Seven additional infants were tested but excluded from subsequent analyses for not completing the experiment due to fussiness. Parents provided written informed consent on behalf of their infants. All procedures were approved by the local ethics committee.

### Procedure and Design

Infants were tested individually in a dimly lit soundproof room. Each infant sat in a high chair or on his/her parent’s lap at a distance of approximately 70 cm from a large projection screen (92.5 cm × 67.5 cm). Parents were instructed to keep their eyes closed and to refrain from interacting with their infants during the study, except for soothing them if they became distressed. Each infant’s looking behavior was recorded for later coding using a concealed camcorder placed beneath the projection screen. Video feed was transmitted directly to a computer in an adjoining room where an experimenter monitored the session remotely.

Each infant received the mental rotation task followed by the object preference task.

#### Mental Rotation Task

Two image streams were presented simultaneously on the left and right sides of the screen (61 cm, 41° of visual angle apart) against a gray background. Each stream consisted of a single two-dimensional Tetris-like figure (9 cm × 7.5 cm; 7.5° × 6° of visual angle), appearing in various orientations across the trial. The stimulus was presented at each orientation for 500 ms, followed by a blank screen lasting 300 ms (see Figure 1). On every third presentation of the stimulus, the image in one stream (the “mirror stream”) depicted the mirror image of the figure presented in the other stream (the “non-mirror stream”). However, the orientation of the stimulus in the mirror stream was always identical to the orientation of the non-mirror stimulus. Different orientations were created by rotating the stimulus in increments of 14° along the picture plane. Orientations were presented in a random order throughout each trial with the constraint that orientations could not vary by more than 180° within any given trial. During each of the four 60-s trials, orientations ranged between 0° and 180°, 90° and 270°, 180° and 360°, or 270° and 90°, respectively (randomly ordered). Before each trial, an attention-getter (looming star with sound effect) was presented centrally until the infant oriented to it; the remainder of each trial took place in silence. The position of the mirror stream alternated between the left and right side of the screen across trials, counterbalancing for side on the first trial across infants.

#### Object Preference Task

**FIGURE 2:**
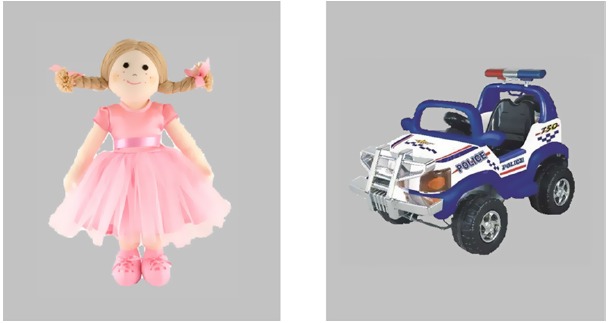
**Stimuli used in the object preference task.** Images of a doll and a toy truck were presented simultaneously on the left and right sides of a frontal screen.

Infants were presented with two 30-s trials of the object preference task. The task included the image of a doll and a toy truck (see Figure 2), each measuring approximately 15.5 cm × 13 cm, presented on the left and right sides of the screen and positioned approximately 61 cm apart. The image of each object subtended approximately 12.5° × 10.5° of visual angle (with a separation of 41° of visual angle between objects). Both objects jittered slightly (∼1.5 cm per 100 ms). As in the mental rotation task, each trial began with an attention-getter (looming star with sound effect). The remainder of the trial took place in silence. Left/right position alternated across the trials, with initial side counterbalanced across infants.

#### Video Coding

High quality videos of each infant were saved digitally. A trained observer blind to the experimental stimuli coded all videos at a frame rate of 30 frames per second. A random sample (25%) of videos was coded by a second observer. Inter-observer reliability was high for both tasks (Pearson’s *r*s > 0.9).

## Results

### Mental Rotation Task

To account for variability in overall attention, we calculated the proportion of time infants spent looking to the mirror stream as a function of their total looking time to both streams [i.e., mirror stream/(mirror stream + non-mirror stream)] across the four trials. Scores greater than 0.50 indicate a preference for the mirror stream relative to the non-mirror stream. In an initial analysis, we found that infants preferred the mirror stream (*M* = 0.55, SD = 0.06) significantly more than would be expected by chance, *t*(55) = 6.25, *p* < 0.001 (*d* = 0.84), with the majority of infants showing this pattern (47/56, *p* < 0.001, binomial test). In a separate analysis, we examined whether performance on this task varied as a function of age; a correlation analysis revealed no significant relation between the age of the infants and their preference for the mirror stream, *r*(54) = –0.05, *p* = 0.716. These analyses show that across the age range tested infants exhibited greater interest in the stream containing the mirror image, suggesting that they recognized the novelty of the mirror stimulus.

**FIGURE 3:**
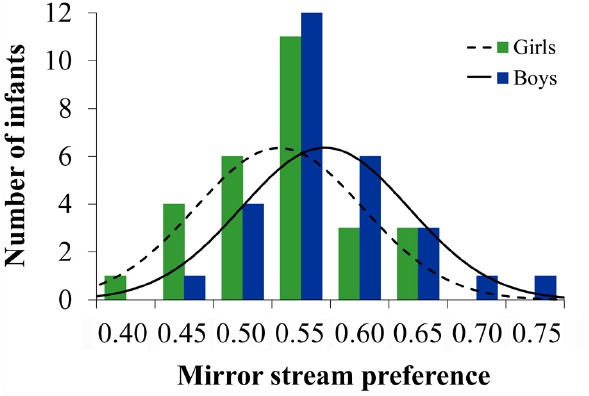
**Histogram with Gaussian distribution of mental rotation performance for boys and girls.** Mirror stream preference scores represent the mean proportion of looking time toward the mirror stream relative to overall looking time. Mirror stream preference scores above 0.50 reflect greater looking toward the mirror stream compared to the non-mirror stream (chance performance = 0.50).

To compare the performance of girls and boys on this task, we conducted a two-way analysis of variance (ANOVA) with the between-subjects variables of sex and side of mirror stream on the first trial. The dependent variable was the proportion of looking time to the mirror stream. This analysis revealed a significant main effect of sex, *F*(1,52) = 4.16, *p* = 0.046 (${\text{η}}_{p}^{2}$ = 0.07), but no other main effect (*p* > 0.2) or interaction (*p* > 0.4). As a group, boys showed greater sensitivity to the mirror stream, spending an average of 3.41% more time looking to the mirror stream than did girls. This finding is consistent with others that show infant boys display an advantage in mental rotation (e.g., Moore and Johnson, 2008; Quinn and Liben, 2008), at least when the task requires distinguishing mirror images. Further analyses suggested that this sex difference could not be attributed to a sex difference in infants’ interest in the task; boys (*M* = 102.16 s, SD = 27.01 s) and girls (*M* = 99.26 s, SD = 38.05 s) exhibited comparable overall looking times (i.e., mirror + non-mirror) across the four trials, *t*(27) = 0.33, *p* = 0.743 (*d* = 0.09). Despite the reported sex difference in mental rotation task performance, additional comparisons of infants’ mirror stream preferences revealed that both boys (*M* = 0.57, SD = 0.06), *t*(27) = 6.01, *p* < 0.001 (*d* = 1.15), and girls (*M* = 0.54, SD = 0.06), *t*(27) = 3.09, *p* < 0.01 (*d* = 0.59), looked longer to the mirror stream than would be expected by the chance level of 0.50 and that the distribution of scores across the two sexes was largely overlapping (see Figure 3). Thus, across sexes, infants were able to recognize the novel rotation within the mirror stream, but boys showed greater discrimination on the task.

### Object Preference Task

For this task, we first compared infants’ looking times to the truck versus the doll. Across the two trials, infants looked significantly longer to the doll (*M* = 22.00 s, SD = 8.51) compared to the truck (*M* = 14.99 s, SD = 6.69), *t*(55) = 4.95, *p* < 0.001 (*d* = 0.92), with the majority of infants showing this pattern (42/56, *p* < 0.001, binominal test), as would be expected given previous work showing infants prefer stimuli containing faces (e.g., Serbin et al., 2001; Jadva et al., 2010). We then analyzed infants’ relative preference for the stimuli by calculating the proportion of time infants spent looking to the toy truck as a function of their total looking time to both objects [i.e., truck/(truck + doll)] across both trials. We found that infants looked significantly more to the doll and less to the truck (*M* = 0.41, SD = 0.13), *t*(55) = 5.24, *p* < 0.001 (*d* = 0.69). A correlation analysis revealed that this effect did not vary within the age range tested, *r*(54) = 0.16, *p* = 0.239.

Although both boys (*M* = 0.44, SD = 0.13), *t*(27) = 2.27, *p* = 0.031 (*d* = 0.46), and girls (*M* = 0.37, SD = 0.12), *t*(27) = 5.49, *p* < 0.001 (*d* = 1.08), showed significantly longer looking to the doll relative to the truck, we examined whether the size of this preference differed for boys and girls by conducting an ANOVA with sex as the between-subjects variable. Side of image presentation (truck/doll) on the first trial was also included as a between-subjects variable. This analysis revealed a significant main effect of sex, *F*(1,52) = 4.69, *p* = 0.035 (${\text{η}}_{p}^{2}$ = 0.08) but no effect of side (*p* > 0.7) and no interaction between the two variables (*p* > 0.3). Again, this sex difference could not be attributed to differences in task engagement, as boys (*M* = 36.14 s, SD = 11.25 s) and girls (*M* = 33.77 s, SD = 9.87 s) exhibited comparable overall looking times (i.e., doll + truck) across the two trials, *t*(27) = 0.74, *p* = 0.460 (*d* = 0.22).

Consistent with extant findings, our findings confirm that both boys and girls at this age prefer the doll over the truck, but, as a group, boys exhibit this effect less strongly than girls (e.g., Serbin et al., 2001; Jadva et al., 2010).

### Does Mental Rotation Relate to Object Preference?

**FIGURE 4:**
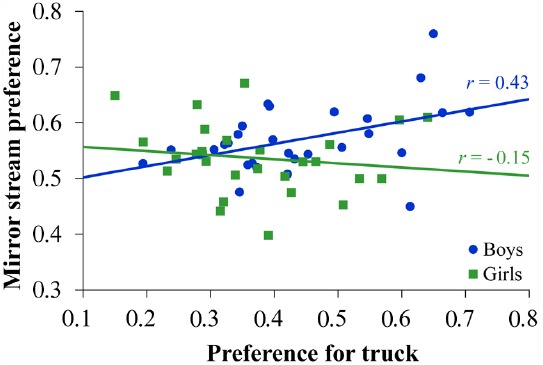
**Scatterplot of the relationship between object preference and mental rotation performance.** Preference for the truck represents infants’ mean proportional looking time to the toy truck relative to overall looking toward either stimulus (0.50 indicates equivalent looking toward the truck and doll). Mirror stream preference scores reflect mean proportional looking time toward the mirror stream relative to overall looking time (0.50 indicates equivalent looking toward the mirror and non-mirror stream). Boys showed a significant positive correlation between object preference and mental rotation performance (*r* = 0.43, *p* < 0.05), whereas the correlation within girls was not statistically significant. Moreover, the correlation within boys was statistically greater than that for girls.

To address this question, we correlated proportion of looking time to the mirror stream on the mental rotation task with the proportion of looking time to the toy truck on the object preference task. We first conducted this analysis at the group level and found a marginally positive correlation, *r*(54) = 0.22, *p* = 0.112. Because the analyses above revealed sex differences on each task, we also conducted correlation analyses by sex. These analyses yielded a significant positive correlation for boys, *r*(26) = 0.43, *p* = 0.022, but not girls, *r*(26) = –0.15, *p* = 0.462 (see Figure 4). A direct comparison revealed that these correlations differed significantly from each other, Fischer *r*-to-*z* test = 2.15, *p* = 0.032. Boys who looked relatively longer to the truck on the object preference task showed proportionally greater looking to the mirror stream on the mental rotation task. In contrast, there was no such relationship for girls. Although performance on neither task was related to age in the above analyses, we examined whether these correlations varied by age. Controlling for age, the effects remained the same, boys: *r_p_*(25) = 0.43, *p* = 0.027; girls: *r_p_*(25) = –0.14, *p* = 0.482. Thus, across the age range tested, object preference was related to mental rotation performance for boys but not for girls.

Could the lack of correlation between mental rotation and object preference in girls be due to less variability in performance on one or both tasks in comparison to boys? To address this, we compared the range of variability on each task for boys and girls. This comparison revealed that boys and girls were comparably variable (mental rotation task: girls, SD = 0.06; boys, SD = 0.06; object preference task: girls, SD = 0.12; boys, SD = 0.13), such that the sex differences in the relation between mental rotation and object preference could not be accounted for by differences in variability on either task.

## Discussion

The aim of the current study was to explore the relationship between mental rotation ability and object preference, two domains in which sex differences have been reported in infancy (e.g., Moore and Johnson, 2008; Jadva et al., 2010). Our results point to similarities and differences between male and female infants in mental rotation performance and object preference, as well as an effect of sex in the relation between these two domains. We discuss these findings below.

### Mental Rotation

Male infants showed an advantage in mental rotation performance on the change detection task. These results align with prior findings from other infant paradigms, providing convergent evidence for a sex difference in mental rotation that emerges early in development (e.g., Moore and Johnson, 2011; Quinn and Liben, 2014). Unlike these previous studies, however, we found that both sexes performed above chance, suggesting that girls, like boys, are capable of representing the orientation of a two-dimensional figure and discriminating mirror images. Moreover, although boys as a group performed better than girls on the mental rotation task, the distributions of scores for boys and girls were largely overlapping, suggesting similarity in the individual differences that characterize performance for the two sexes.

A caveat to these findings is that they rely on the assumption that greater looking toward the mirror stream in our change detection paradigm reflects mental rotation. More specifically, we have accepted that a preference for the mirror stream indicates that infants formed a mental representation of the target object (the Tetris-like figure) and its rotational movement, and consequently, that they recognized the novelty of the mirror reversal. There could be alternative strategies, however. In particular, because the figure used in the task was presented from multiple orientations, infants may have represented its shape independent of a specific orientation. Such a representation would be considered orientation invariant or viewpoint independent (Biederman and Gerhardstein, 1993) and could allow infants to recognize the novelty of the mirrored figure without representing its rotational movement. Although we cannot rule out this alternative directly, the fact that infants were capable of mirror discrimination at an above chance rate argues against this. Viewpoint-independent representations are often associated with difficulty discriminating mirror images (Farah and Hammond, 1988; Vanrie et al., 2002). It is also unclear how the sex difference reported in this and previous studies would align with such a strategy. Nevertheless, future research would do well to consider alternative strategies to mental rotation in visual attention paradigms with infants (cf. Möhring and Frick, 2013).

### Object Preference

Both boys and girls exhibited greater preferences for the doll compared to the toy truck. However, boys showed relatively greater visual attention to the toy truck than did girls. These results are consistent with extant findings showing that, beginning in infancy, both male and female infants prefer objects with faces over those without, but that boys show greater interest in toy vehicles than do girls (e.g., Serbin et al., 2001; Jadva et al., 2010). Moreover, these early sex differences in visual interest parallel those found in play preferences later in childhood (Pasterski et al., 2005), suggesting that later play preferences may build on early object preferences or that shared mechanisms account for both sexually dimorphic object and play preferences (Alexander et al., 2009).

One concern with this task is that we used stimuli that were sex-typed not only in content (doll vs. toy truck) but also in color (pink vs. blue, respectively), making it unclear what factor drove the preference. It should be noted, however, that younger children and infants do not exhibit the sex-typed color preferences observed later in development (Jadva et al., 2010; LoBue and DeLoache, 2011). We would thus suggest that the sex difference reported here likely relates specifically to the content of the objects.

### Relationship between Mental Rotation and Object Preference

In addition to the findings above, we found that, among boys, greater visual interest in a toy truck compared to a doll was associated with higher performance on our mental rotation task, as measured by longer looking to the mirror stream. In contrast, we found no such association among girls. This sex difference in the relation between mental rotation and object preference held across the age range tested and did not reflect sex differences in variability on the two tasks.

Using a comparatively large sample of infants, we have replicated findings from previous studies that have reported early sex differences in mental rotation and object preference (e.g., Quinn and Liben, 2008; Alexander et al., 2009). In the Introduction, we described two prevalent accounts of sex differences in mental rotation, and others also have offered accounts for early, and perhaps even innate, sex differences in object preference (see Alexander et al., 2009). Here, we offer accounts for the novel finding that, at least in infancy, the association between mental rotation and object preference varies by sex such that boys show an association whereas girls do not.

One possible account for the sex difference in the relationship between mental rotation performance and object preference is that it reflects a difference in perceptual biases between boys and girls (Alexander et al., 2009). It has been suggested that males are innately more attentive to motion and objects that afford motion than are females (Alexander, 2003; Benenson et al., 2011). Studies with non-human primates (Alexander and Hines, 2002; Hassett et al., 2008) and human neonates (Connellan et al., 2000) documenting a male preference for objects with affordances for propulsive movement are consistent with innate, evolutionarily-based proclivities toward these objects (Moller and Serbin, 1996). The relation between mental rotation and a preference for toy vehicles could thus reflect that a proclivity for motion or motion-producing objects is more common among boys than girls, and this perceptual preference may promote the development of mental rotation abilities specifically in young boys. More specifically, infants with a perceptual preference for motion may spend more time engaging with motion-producing objects, leading to greater experience with the manual rotation of objects and greater mental rotation abilities as a result. This argument is supported by previous findings showing that infants’ mental rotation performance is enhanced by physically interacting with the rotating objects of interest (Möhring and Frick, 2013; Frick and Wang, 2014), which suggests that hands-on experience with motion-producing objects may scaffold the development of mental rotation in early childhood.

Another possibility is that the sex difference in the relation between mental rotation and object preference reflects the common influence of sexually dimorphic prenatal androgen exposure in performance on both types of tasks (Alexander, 2003; Hines, 2004). Research has shown that early androgen levels represent one mechanism through which individual and sex differences in toy preferences and mental rotation ability develop (Berenbaum and Hines, 1992; Grimshaw et al., 1995; Lamminmäki et al., 2012). Following from this, early androgen levels could relate to better mental rotation ability and male-typed object preferences in boys, but not girls, because typically developing girls may not be exposed to the levels of androgens necessary to alter the development of related neural systems that underlie both types of cognition.

These two accounts are not mutually exclusive, however. The organizational effect of early androgens within the visual system may lead to sexually dimorphic visual processing of the perceptual features associated with motion and characteristic of male-typed objects, which could give rise to sex differences in play preferences (Alexander and Hines, 2002) that promote the development of mental rotation abilities. Early androgens may alter the time course of the development of the magnocellular and parvocellular visual pathways, leading to sex-specific specialization of the dorsal and ventral processing streams (Alexander, 2003; see also Alexander and Hines, 2002). The dorsal stream receives input mostly from the magnocellular pathway and shows specialization for object location and motion. The ventral stream, in contrast, receives input mostly from the parvocellular pathway and shows specialization for object recognition. There is evidence that the dorsal stream develops prior to the ventral stream, and research in non-human animals suggests that early androgens modulate the development of visual and neural structures related to the two streams (Bachevalier and Hagger, 1991; Salyer et al., 2001). If sexual dimorphism in visual pathway development occurs in humans as well, as vision research in human infants has suggested (Bauer et al., 1986; Gwiazda et al., 1988), then males may be more perceptually attuned to the motion of objects due to later maturation within the parvocellular pathway (cf. Bachevalier and Hagger, 1991), whereas females may be more perceptually attuned to the static features of objects, such as form and color. Consequently, males may exhibit greater interest in motion and motion-producing objects, which, in turn, may enhance their mental rotation abilities.

An alternative interpretation of the observed relation between mental rotation and object preference for boys, but not girls, rests on sex-specific experiences present from early in development. In particular, the preference observed for the toy truck could reflect greater familiarity with this type of object or other spatially relevant toys in boys compared to girls. Parents provide children with gender-congruent toys beginning in the first 2 years of life (Pomerleau et al., 1990), and male-typical toys often encourage more spatially relevant play (Baenninger and Newcombe, 1989). Toy vehicles, unlike dolls, are symmetrically interesting (i.e., front and back) and capable of propulsive motion, meaning that play with these objects may encourage the encoding of information related to orientation and the prediction of future object trajectories. Consequently, exposure to such toys may heighten one’s sensitivity to spatial relations and, in turn, promote the development of mental rotation abilities. On this view, preferential looking toward the toy truck may have reflected greater experience with objects that encourage spatially relevant types of play, promoting visuospatial thinking and leading to a male advantage in mental rotation at this early age. The challenge for this interpretation, however, is that infants’ exposure to spatially relevant toys and activities is limited. Children within the age range tested here have likely had minimal experience with spatially relevant play. Furthermore, it is unlikely that sex-typed toy preferences result exclusively from environmental factors, as findings in typically-developing children, clinical populations, and non-human primates suggest biological determinants for sex-typed object preferences (Alexander and Hines, 2002; Pasterski et al., 2005; Lamminmäki et al., 2012).

A common thread among the interpretations of the current findings is a possible role for early object preference in the development of mental rotation ability, which parallels the view in the literature examining the association between spatial activities and visuospatial aptitude (Baenninger and Newcombe, 1989; Voyer et al., 2000). Although there are logical reasons for assuming that exposure to certain objects and activities affects mental rotation performance, it is possible that mental rotation ability or visuospatial aptitude more generally influences preferences for specific objects and activities. Given the correlational design of our study, we acknowledge that mental rotation ability could influence object preference or that this relationship could be bidirectional. Further research is needed to distinguish these possibilities in the context of the sex-specific association between mental rotation and object preference reported in this study. Moreover, in light of growing evidence that mental rotation abilities are related to math performance early in childhood (e.g., Gunderson et al., 2012; Cheng and Mix, 2014) and that sex differences in mental rotation may contribute to the gender gap in STEM achievement observed later in development (e.g., Casey et al., 1997; Ganley et al., 2014), future research should consider the influence of the sex-specific relation between object preference and mental rotation on broader associations between sex, visuospatial aptitude, and STEM success.

## Author Contributions

Research design: SL, HU, SJ; Data collection: HU, SJ; Data analysis: SL, JL; Manuscript preparation: JL, SL.

### Conflict of Interest Statement

The authors declare that the research was conducted in the absence of any commercial or financial relationships that could be construed as a potential conflict of interest.
